# Suppression of ribosomal protein synthesis and protein translation factors by Peg-interferon alpha/ribavirin in HCV patients blood mononuclear cells (PBMC)

**DOI:** 10.1186/1479-5876-10-54

**Published:** 2012-03-22

**Authors:** Rahul Gupta, Sun Kim, Milton W Taylor

**Affiliations:** 1 School of Informatics and Computing, Indiana University, Bloomington, IN, USA; 2 Department of Biology, Indiana University, Bloomington, IN, USA; 3 Department of Computer Science and Engineering, Bioinformatics Institute, Interdisciplinary Program in Bioinformatics, Seoul National University, Seoul, Korea

**Keywords:** Ribosomal proteins, Transcription factors, Interferon, Ribavirin, Hepatitis C

## Abstract

**Background:**

We have previously reported the induction of many interferon stimulated genes (ISGs) in PBMC collected from patients infected with HCV at various times after initiation of interferon-ribavirin treatment using DNA microarrays to identify changes in gene expression with time. Almost as many genes are down regulated (suppressed) during interferon-ribavirin treatment as are up regulated.

**Methods:**

DNA microarrays were analyzed by different software, including MAS5 (Affymetrix-Kegg) and GSEA (gene set enrichment analysis) to identify specific pathways both up regulated and down regulated. Data was assessed from a clinical trial, which was a microarray analysis from 68 patients.

**Results:**

Up regulated genes included genes associated with NF-kb, toll like receptor cytokine -cytokine interaction, and complement and adhesion pathways. The most prominent pathway down regulated was that for ribosomal structural proteins, and eukaryotic translational factors. Down regulation of ribosomal protein genes continued through the treatment up to the last measurement, which was at day 28.

**Conclusions:**

This suppression of the protein synthetic apparatus might explain the long-term side effects of interferon-ribavirin, and explain a non-specific effect of interferon-ribavirin on viral protein synthesis. There was no evidence for unique transcription factors or micro RNA involvement.

## Introduction

Hepatitis C virus (HCV) infection is a significant global public health problem, affecting approximately 150 million individuals worldwide and over 4 million in the United States alone, where it is the predominant blood-borne infection [1]. It is currently the leading indication for a liver transplant and is responsible for 8,000-10,000 deaths annually. Treatment with Interferon -α has formed the backbone of therapy against HCV, first as mono-therapy, then in combination with the nucleoside analogue ribavirin. Current standard of care for chronic HCV infection consists of a regimen of pegylated interferon in combination with ribavirin and more recently the addition of a protease inhibitor [2]. The combination of pegylated IFN-alpha and ribavirin successfully eradicates the virus from 50-60% of those treated depending on antigenic strain of the virus and race of the patient. Genotype 1a and 1 b the most prevalent genotypes in the USA are the most difficult to eradicate, and the sustained response rate among African Americans is lower than that found in other racial groups [3].

Conventional analysis of DNA microarrays between control (before treatment) and interferon treated samples results in large lists of genes induced or down regulated that in many cases appear to be unconnected [4,5]. In the typical experiment one looks for fold increases in expression of specific genes, using paired Student's *t*-test to assess significance between the different treatments (or classes). We have reported that approximately 1,000 genes are modified in gene expression in RNA isolated from PBMC of hepatitis C patients being treated with Pegasys/ribavirin [5] using a 1.5 fold cut off and a p value of < 0.001. Utilizing a mathematical approach in which we compared clustering of patient response with gene expression we have identified 36 genes that appear to be closely correlated with the antiviral response [6] An alternative method of analyzing microarrays, called gene set enrichment analysis (GSEA) has been published. This approach utilizes our knowledge of specific pathways, chromosomal location, presence of common DNA sequences and different transcription binding sites, and instead of giving each gene a statistical value it determines whether members of a gene set *S *tend to occur toward the top (or bottom) of the list *L*, which will correlate with phenotype. This approach has been successfully used in analyzing data from muscle biopsies from diabetics vs. healthy controls [7]. The method revealed that genes involved in oxidative phosphorylation (OXPHOS) show reduced expression in diabetics, although the average decrease per gene is only 20%. Thus this method can identify pathways that may be important, since a 20% increase or decrease in specific metabolic pathways may affect the outcome of many disease situations and might not be detected by the software used to analyze microarrays, which usually is set at a two-fold increase or decrease and a p value of < 0.001. In our previous work we have emphasized as have others the role of gene induced following interferon/ribavirin treatment but ignored genes suppressed or down regulated

In this paper we present a reanalysis of data from a large clinical trials, Virahep C, in which DNA microarrays were performed on PBMC from 68 patients receiving a regimen of interferon-alpha 2a (Pegasys)/ribavirin. In particular we noted the suppression of gene transcription in the ribosomal proteins-eukaryotic translation and elongation factor pathways.

## Materials and methods

### Patient treatment and samples

The patient samples used in this study have previously been described [5]. Approval was received from the Indiana University Human Subjects Committee.

### RNA extraction

Peripheral Blood Mononuclear Cell (PBMC) Preparation and RNA extraction were as previously described [5,8,9].

### RNA labeling and hybridization

Preparation of cDNA, cRNA, and labeling were carried out according to the protocols recommended by Affymetrix in the GeneChip^® ^Expression Analysis Technical Manual (Affymetrix, Santa Clara, CA), as previously described [9].

### Array analysis and data processing

The Human Genome U133A microarray containing 22,000 genes was used. The microarrays were scanned using a dedicated Model 3000 scanner, controlled by Affymetrix Microarray Suite 5 software (MAS5). Global scaling to a target intensity of 1,000 normalized the average intensity on each array. Data were exported from MAS5 into a custom-designed database (MicroArray Data Portal) at the Center for Medical Genomics (IUPUI, Indianapolis). For GSEA analysis no filters were applied to the gene sets, thus all 22,000 genes at each time point were used for this analysis

### GSEA analysis

We used the GSEA software [10] to identify pathways that appeared to be over expressed in one or other of our phenotypic classes. GSEA evaluates a query microarray dataset using a collection of gene sets. The Q value for determining significance was an FDR of < 0.25

## Results

We have previously reported that the transcription of a large number of genes is induced or increased above background in PBMC harvested from patients with hepatitis C during the course of treatment with pegylated-Interferon-alpha (Pegasys) and ribavirin [5,8] Although the transcription of an equal number of genes is down-regulated we have not previously attempted to classify them by pathway analysis. We have reanalyzed this data using alternative methods of analysis including GSEA [10], and other methods that classify genes by pathways rather than as individual genes. The results of GSEA analysis are presented in Table 1 using a False Detection Rate (FDR) calculation of < 0.025 for both pathways up regulated and down regulated, using the latest Kegg and Reactome classification. The number of pathways induced declines by day 7 reflecting the return to near normal levels of gene transcription within a week of initiating treatment and the transient nature of much of the interferon response [8]. Day 28 was the last day in which data was available. The major pathways induced are DNA sensing pathways and the RIG-I like receptor signaling pathway reflecting binding of dsRNA to toll -like receptors. The only pathway down regulated at the level of FDR, 0.25 in this group of patients following treatment is the ribosomal pathway including ribosomal proteins initiation factors and elongation factors required for protein synthesis. The down regulated genes and fold difference following treatment at day 1 and 2 are presented in Table 2 and Table 3.

**Table 1:** PBMC treated with IFN/ribavirin.

Day 1 enrichment	# of genes	
**Pathway**	**SIZE**	**FDR q-val**

KEGG_CYTOSOLIC_DNA_SENSING_PATHWAY	31	0.00

KEGG_RIG_I_LIKE_RECEPTOR_SIGNALING_PATHWAY	40	0.00

KEGG_PROTEASOME	41	0.02

KEGG_TOLL_LIKE_RECEPTOR_SIGNALING_PATHWAY	70	0.02

KEGG_LYSOSOME	88	0.04

KEGG_TRYPTOPHAN_METABOLISM	17	0.04

KEGG_COMPLEMENT_AND_COAGULATION_CASCADES	21	0.06

KEGG_CITRATE_CYCLE_TCA_CYCLE	25	0.09

KEGG_CHEMOKINE_SIGNALING_PATHWAY	104	0.15

KEGG_LEISHMANIA_INFECTION	51	0.15

KEGG_SNARE_INTERACTIONS_IN_VESICULAR_TRANSPORT	27	0.21

KEGG_APOPTOSIS	64	0.20

KEGG_PPAR_SIGNALING_PATHWAY	26	0.20

Day 1 down regulated		

KEGG_RIBOSOME	76	0.14

Day 2 enrichment		

KEGG_RIG_I_LIKE_RECEPTOR_SIGNALING_PATHWAY	40	0.00

KEGG_CYTOSOLIC_DNA_SENSING_PATHWAY	31	0.00

KEGG_TOLL_LIKE_RECEPTOR_SIGNALING_PATHWAY	69	0.00

KEGG_LYSOSOME	88	0.02

KEGG_COMPLEMENT_AND_COAGULATION_CASCADES	23	0.04

KEGG_PROTEASOME	41	0.06

KEGG_CHEMOKINE_SIGNALING_PATHWAY	103	0.08

KEGG_CYTOKINE_CYTOKINE_RECEPTOR_INTERACTION	95	0.12

KEGG_NOD_LIKE_RECEPTOR_SIGNALING_PATHWAY	37	0.15

KEGG_SNARE_INTERACTIONS_IN_VESICULAR_TRANSPORT	27	0.15

KEGG_APOPTOSIS	65	0.19

Day 2 down regulated		

KEGG_RIBOSOME	76	0.15

Day 7 enrichment		

KEGG_RIG_I_LIKE_RECEPTOR_SIGNALING_PATHWAY	39	0.00

KEGG_CYTOSOLIC_DNA_SENSING_PATHWAY	30	0.00

KEGG_TOLL_LIKE_RECEPTOR_SIGNALING_PATHWAY	66	0.09

KEGG_APOPTOSIS	61	0.15

Day 7 down regulated		

KEGG RIBOSOME	76	0.20

Day 14 enrichment		

KEGG_RIG_I_LIKE_RECEPTOR_SIGNALING_PATHWAY	39	0.00

KEGG_CYTOSOLIC_DNA_SENSING_PATHWAY	31	0.00

KEGG_TOLL_LIKE_RECEPTOR_SIGNALING_PATHWAY	67	0.13

KEGG_COMPLEMENT_AND_COAGULATION_CASCADES	21	0.18

KEGG_PROTEASOME	41	0.22

Day 14 down regulated		

KEGG_RIBOSOME	76	0.18

Day 28 enrichment		

KEGG_RIG_I_LIKE_RECEPTOR_SIGNALING_PATHWAY	38	0.00

KEGG_CYTOSOLIC_DNA_SENSING_PATHWAY	30	0.00

Day 28 down regulated		

KEGG_RIBOSOME	76	0.25

68 patients

**Table 2:** List of ribosomal genes down regulated at day 1 and day 2 with fold difference.

Ribosomal protein	Day 1	Day 2
RPL10	-1.67	-1.45

RPL10	-1.58	-1.47

RPL10A	-1.60	-1.52

RPL13	-1.87	-1.65

RPL13	-1.75	-1.57

RPL13	-1.58	-1.48

RPL13	-1.49	-1.46

RPL13	-1.50	-1.41

RPL13A	-1.53	-1.41

RPL15	-1.60	-1.46

RPL18	-1.61	-1.51

RPL18A	-2.06	-1.52

RPL22	-1.79	-1.63

RPL27AP	-1.68	-1.44

RPL29	-1.87	-1.50

RPL29	-1.71	-1.45

RPL3	-2.13	-1.74

RPL3	-1.62	-1.53

RPL3	-1.58	-1.50

RPL3	-1.57	-1.46

RPL35	-1.37	-1.36

RPL35A	-1.58	-1.37

RPL36	-1.53	-1.31

RPL4	-1.78	-1.72

RPL4	-1.83	-1.77

RPL4	-1.61	-1.56

RPL4	-1.83	-1.77

RPL6	-1.42	-1.35

RPL8	-1.71	-1.52

RPLP0	-1.89	-1.65

RPLP0	-1.47	-1.37

RPS10L	-1.53	-1.48

RPS14	-1.90	-1.67

RPS16	-1.57	-1.48

RPS19	-1.52	-1.32

RPS19	-1.45	-1.30

RPS2	-1.80	-1.34

RPS2	-1.60	-1.43

RPS28	-1.69	-1.54

RPS3	-1.52	-1.35

RPS5	-2.02	-1.82

RPS6	-1.55	-1.47

RPS6	-1.41	-1.38

RPS7	-1.45	-1.35

RPS8	-1.56	-1.55

RPS9	-1.46	-1.33

RPS9	-1.47	-1.37

(average from 68 patients including non-responders

**Table 3:** Elongation and Initiation factors down regulated

Elongation factor	Day 1	Day 2
EEF1B2	-1.69	-1.69

EEF1D	-1.67	-1.53

EEF1G	-1.68	-1.62

EEF1G	-1.68	-1.61

EEF1G	-1.68	-1.62

EEF2	-1.95	-1.76

EEF2	-1.70	-1.54

**Initiation factor**		

EIF2AK3	-1.50	-1.41

eIF3k	-1.30	-1.29

eIF3k	-1.26	-1.28

eIF3k	-1.18	-1.21

EIF3S5	-1.52	-1.53

EIF3S6IP	-1.90	-1.87

EIF3S7	-1.38	-1.38

EIF3S8	-1.19	-1.27

EIF4B	-2.20	-1.97

EIF4B	-2.38	-2.07

EIF4B	-1.58	-1.42

EIF5B	-1.31	-1.36

**Figure 1:**
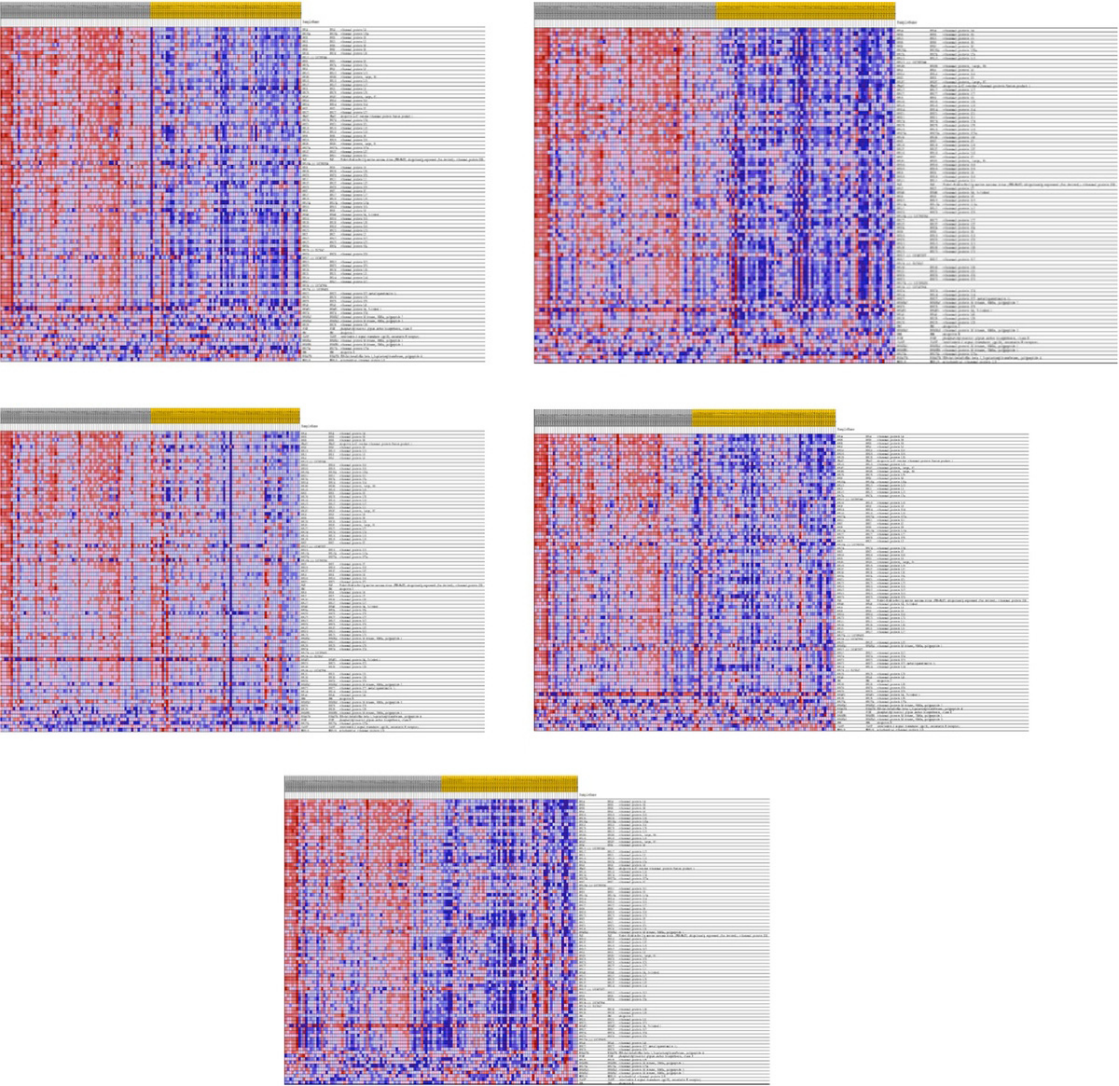
**Heat map of ribosomal protein and translation factor pathway**. Heat map from day 1, 2, 7, 14 and 28 days after treatment initiation.

Figure 1(a-e) present heat map of the ribosomal proteins and other genes related to translation of proteins at the various times during treatment compared to before treatment.

**Figure 2:**
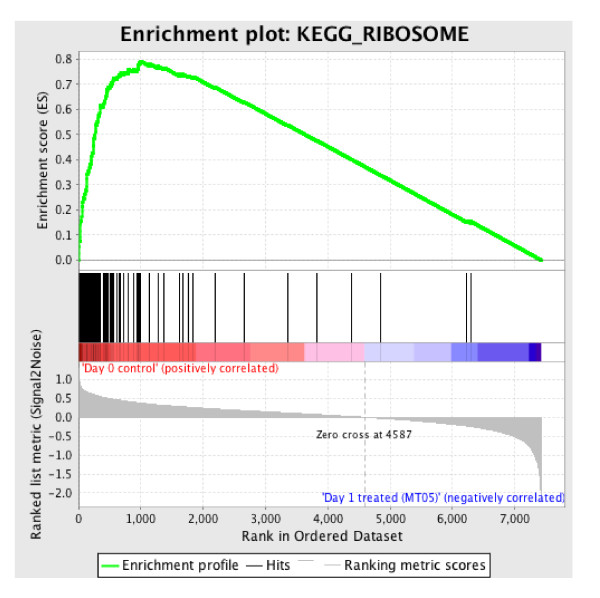
**Enrichment plot of Ribosomal proteins and translation factors at day 1 after initiation of treatment**.

Figure 2 presents the enrichment data for ribosomal proteins and translation factors. A cluster at the top or bottom of the graph indicates significance. Unlike many other pathways, the suppression of ribosomal proteins and translation factors remains constant through out the treatment period. Although it is weaker at day 7, 14 etc., when much of the interferon/ribavirin effect has worn off.

## Discussion

Treatment of HCV patients with pegylated interferon and ribavirin results in a suppression of expression of mRNA for ribosomal proteins and factors involved in protein synthesis, in PBMC isolated from patients. This suppression is global for ribosomal proteins and eukaryotic translation factors and persists through out the treatment period up to 28 days. The extent of down regulation is approximately 50%.

We have previously reported the down regulation of a few individual ribosomal proteins and translation factors in PBMC in vitro and in vivo [5,9] following treatment with Interferon/ribavirin. In this paper we show that this is a global effect on the transcription of ribosomal protein genes. We have examined the effect of ribavirin alone on PBMC in vitro and find some decrease in mitochondrial ribosomal protein transcription but not on cytoplasmic ribosomal proteins (data not shown). Jiang et al. [11] reported the suppression of ribosomal protein L23 transcription during growth of a melanoma in culture treated with interferon alpha, beta and gamma but in the absence of ribavirin.

Meier et al. 2003 [12] reported the inhibition of DNA, RNA and protein synthesis in vitro on PBMC treated with PHA in the presence of high doses of ribavirin. This was a general effect and could be reversed by the addition of guanosine. A thorough analysis by Wadell et al. [13] shows a few ribosomal protein genes as being down regulated by IFN-beta and IFN-gamma in PBMC, although not by IFN-alpha. However the fold cut off used in these experiments as in our earlier experiments would not pick up the whole pathway. Thus it is likely that the combination of Interferon and ribavirin specifically affects transcription of the protein synthetic apparatus.

Unlike other genes affected by interferon/ribavirin treatment where we see transient changes in ISGs 6 days after interferon treatment, the down regulation of transcription of ribosomal proteins appears to be quite constant.

Interferon by itself has previously been shown to inhibit protein synthesis in a specific manner. This is through the dsRNA pathway in which RNAse L is activated and degrades viral RNA [14,15]. However it is unlikely that this down regulation of ribosomal protein gene mRNA is the result of non-specific RNA degradation since there is no effect over all in amounts of specific RNA in the cell as judged by the large number of genes (the majority) not affected by interferon/ribavirin treatment.

PKR an interferon induced (and activated) enzyme has been shown to phosphorylate eukaryotic initiation factor (EIF2) resulting in inhibition of viral protein synthesis [16]. Phosphorylation of eIF2 may have an effect on overall protein synthesis, however this is unlikely the case here since there is no evidence for general inhibition of protein synthesis. The expression of most genes is constant, and only a small percentage are either induced or suppressed. That viral protein synthesis in inhibited is shown by the decrease in viral titer during treatment in responding patients [5]. However the decrease in ribosomal protein gene transcription occurs even in non-responding patients, although to a slightly lower level. This is in keeping with our previous data in which we have shown that in non-responsive patients there is a general blunting of the interferon/ribavirin effect [5].

MicroRNAs have been implicated in regulation of immune response in lymphocytes and in particular miRNA-146a has been shown to inhibit by a feed back mechanism the expression of interferon inducible genes. However a search of known microRNAs has not shown any relationship to ribosomal mRNA or Eukaryotic translation factors. It is possible that unknown microRNAs interact with common sequences in these genes and suppress transcription or degrade these RNAs. However this is purely speculative. We also were unable to find any common transcriptional regulatory sequence upstream of these genes. Previous reports [17-19] have indicated a possible role for c-myc in regulating ribosomal protein transcription, however level of c-myc at the transcription level is unaffected by interferon/ribavirin in our analysis. It is likely that as cells slow down in growth, and undergo apoptosis that the rate of ribosomal protein and other proteins involved in translation will also decrease. We have previously noted (unpublished data) that myc transcription is significantly down regulated in a b-cell line, Daudi following treatment with interferon-alpha. Thus the decrease reported here might reflect a decrease in ribosomal protein transcription in one class of immune cells.

The finding of a general decrease in the protein translation machinery would be an effective way of inhibiting virus. Viral load decrease often occurs in vivo a few days after the initial treatment with interferon/ribavirin. However this drug combination may also partially inhibit host protein synthesis in general, and lead to a variety of side effects in the patients. This could in part be an explanation for the flu like symptoms and anemia often seen in patients undergoing treatment with interferon/ribavirin. Whether this is due to interferon alone, or only occurs in the presence of ribavirin is not clear, although experiments in which PBMC were treated only with ribavirin did not show this effect on ribosomal proteins. However the levels used in these experiments may have been too low. This effect was not found in other cell lines treated with interferon in vitro and thus may be unique to PBMC [20] or to the combination treatment.

## Conclusions

During treatment of hepatitis C patients with interferon/ribavirin there is up regulation and down regulation of transcription of many genes. In this analysis we show that the genes for ribosomal proteins, and eukaryotic transcription and translation factors are down regulated at all time points measured (day 1,2,7,14, and 28). Down regulation of these genes has not been found in other cell types treated with interferon alone in vitro. We suggest that these data may explain in part the side effects often noted following interferon/ribavirin treatment as well as an early inhibition of viral replication.

## Competing interests

The authors declare that they have no competing interests.

## Authors' contributions

RG worked on this project towards a MS degree in informatics: MWT developed the data and helped with the analysis. KS helped with data analysis and statistics, and was mentor to RG. All authors read and approved the final manuscript.
